# *AtMYB72* aggravates photosynthetic inhibition and oxidative damage in *Arabidopsis thaliana* leaves caused by salt stress

**DOI:** 10.1080/15592324.2024.2371694

**Published:** 2024-06-25

**Authors:** Hongrui Zhang, Yinuo Wu, Hongbo Zhang, Nan Sun, Hongjiao Zhang, Bei Tian, Tanhang Zhang, Kexin Wang, Xu Nan, Huiui Zhang

**Affiliations:** a College of Life Sciences, Northeast Forestry University, Harbin, China; b Aulin College, Northeast Forestry University, Harbin, China; c Key Laboratory of Heilongjiang Province for Cold-Regions Wetlands Ecology and Environment Research, Harbin University, School of Geography and Tourism, Harbin, China

**Keywords:** *Arabidopsis thaliana*, salt stress, *AtMYB72*, photosynthesis, antioxidant system

## Abstract

MYB transcription factor is one of the largest families in plants. There are more and more studies on plants responding to abiotic stress through MYB transcription factors, but the mechanism of some family members responding to salt stress is unclear. In this study, physiological and transcriptome techniques were used to analyze the effects of the R2R3-MYB transcription factor *AtMYB72* on the growth and development, physiological function, and key gene response of *Arabidopsis thaliana*. Phenotypic observation showed that the damage of overexpression strain was more serious than that of Col-0 after salt treatment, while the mutant strain showed less salt injury symptoms. Under salt stress, the decrease of chlorophyll content, the degree of photoinhibition of photosystem II (PSII) and photosystem I (PSI) and the degree of oxidative damage of overexpressed lines were significantly higher than those of Col-0. Transcriptome data showed that the number of differentially expressed genes (DEGs) induced by salt stress in overexpressed lines was significantly higher than that in Col-0. GO enrichment analysis showed that the response of *AtMYB72* to salt stress was mainly by affecting gene expression in cell wall ectoplast, photosystem I and photosystem II, and other biological processes related to photosynthesis. Compared with Col-0, the overexpression of *AtMYB72* under salt stress further inhibited the synthesis of chlorophyll a (Chla) and down-regulated most of the genes related to photosynthesis, which made the photosynthetic system more sensitive to salt stress. *AtMYB72* also caused the outbreak of reactive oxygen species and the accumulation of malondialdehyde under salt stress, which decreased the activity and gene expression of key enzymes in SOD, POD, and AsA-GSH cycle, thus destroying the ability of antioxidant system to maintain redox balance. *AtMYB72* negatively regulates the accumulation of osmotic regulatory substances such as soluble sugar (SS) and soluble protein (SP) in *A. thaliana* leaves under salt stress, which enhances the sensitivity of Arabidopsis leaves to salt. To sum up, *MYB72* negatively regulates the salt tolerance of *A. thaliana* by destroying the light energy capture, electron transport, and antioxidant capacity of Arabidopsis.

## GRAPHIC ABSTRACT

**Figure:**
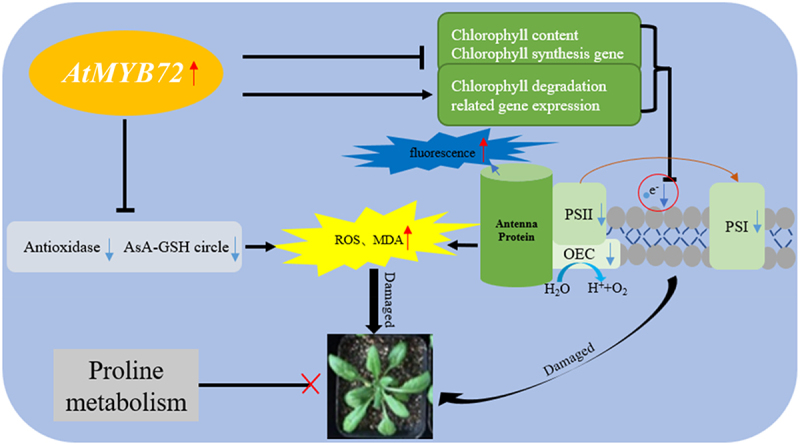

## 1. Introduction

As an abiotic stress, salt stress has adverse effects on plant growth, crop yield, and ecosystem composition, which seriously restricts the development of global agriculture and is also the main reason for abandoning land aquifers for agriculture.^1,2^ High concentrations of Na^+^ and Cl^−^ often lead to salt stress in the soil. Once absorbed by the roots, the ability of plants to absorb water will be reduced, which will have a negative impact on plant growth by damaging metabolic processes and reducing photosynthetic rates.^3,4^ Breeding salt-tolerant crops to optimize salt-contaminated water and soil resources has been a much-sought-after scientific goal but little has been achieved so far. Because it has been found that the main genetic traits that determine salt tolerance in plants are almost absent.^5^ Therefore, it is particularly important to clarify the resistance mechanism of plants and determine how to improve the tolerance of plants to salt stress.

Photosynthesis is a vital biological process necessary for the survival of all biotas. It maintains the normal life activities of plants by synthesizing carbon skeletons to provide raw materials for biological macromolecules. Salinity has a significant impact on photosynthesis, with PSII being especially susceptible to salt stress.^6–8^ Studies have shown that environmental pressures may decrease the photosynthetic ability of plants, perhaps owing to factors such as pigment levels, chloroplast and PSII structure, and enzymes relevant to photosynthetic processes.^9^ In addition, plant photosynthesis is reduced under salt stress, accompanied by the incomplete reduction of oxygen in the photosynthetic electron transport chain or the incomplete oxidation of water to produce a large amount of reactive oxygen species (ROS),^10^ which can cause further damage to plants and even death. ROS primarily consists of O_2_^−•^ (superoxide anion); •OH, (hydroxyl radical); ^1^O_2_, (singlet oxygen) and H_2_O_2_, (hydrogen peroxide).^11^ The photosynthetic electron transport chain (PETC) is the main source of reactive oxygen species in plants. The redox reaction in PETC generates ROS when electron acceptors, such as molecular oxygen, are limited during electron transfer.^12,13^ Excessive ROS in plants is highly toxic and can cause damage to surrounding proteins, lipids, carbohydrates, and DNA, leading to oxidative stress.^14,15^ Plants usually produce a series of physiological and biochemical reactions to establish systemic defense mechanisms to overcome various abiotic stresses at multiple levels.^16^ Plants can reduce oxidative damage by regulating the activity of antioxidants and antioxidant enzymes to better adapt to stressful environments. Most studies have shown that the activities of antioxidant enzymes including superoxide dismutase (SOD), catalase (CAT), and peroxidase (POD) increased significantly under salt stress by removing free oxides, maintaining REDOX balance, and protecting cellular structure. SOD is the first line of defense of the plant antioxidant system, which can convert the accumulated superoxide molecules into oxygen and H_2_O_2_, and then CAT and POD convert H_2_O_2_ into water and oxygen. In addition, under salt stress, the activities of related enzymes in the ascorbic acid (AsA)-glutathione (GSH) cycle were also enhanced. Ascorbate peroxidase (APX), glutathione peroxidase (GPX), glutathione reductase (GR) cooperated with dehydroascorbate reductase (DHAR) and monodehydroascorbate reductase (MDHAR) to reduce the production of lipid peroxidation product malondialdehyde (MDA) and maintain the stability of cell membrane structure.^17–20^ At the same time, plants also trigger osmotic regulators to maintain redox balance, such as soluble sugar (SS), soluble protein (SP), proline (Pro), etc. It has been reported that salt stress can lead to a large accumulation of Pro content.^21,22^ In conclusion, plants use antioxidant enzymes and osmoregulatory substances to reduce oxidative damage caused by H_2_O_2_, limit cell lipid peroxidation, and improve cell stress resistance.^21,23,24^

The transcriptional level of downstream stress response-related genes are often used as an important indicator to evaluate stress intensity and plant tolerance.^25^ Transcription factors (TFs) play an important role as central regulators, linking the perception and transmission of signals generated under stress with the response of downstream genes. In complex environments, TFs can perform specific functions to enhance plant adaptability through specific molecular dynamics methods such as homodimerization and heterodimerization, as well as post-translational modification.^26^ Studies have shown that a variety of transcription factors have outstanding contributions in promoting plant growth and development, reducing oxidative damage, and enhancing salt stress tolerance, such as WRKY^27^、SPL^28^、MYB^29^、bZIP^30^、AP2/ERF.^31^ MYB transcription factor is one of the largest transcription factor superfamilies, which plays a regulatory role in plant development and defense response.^32^ The first plant MYB gene *C1* was isolated from maize (*Zea mays* L.) in 1987. It encodes a c-MYB-like transcription factor involved in anthocyanin biosynthesis.^33^ Subsequently, numerous experiments have found that MYB family transcription factors are involved in the synthesis of primary and secondary metabolites.^34^ EsMYB9 in Epimedium is involved in the regulation of flavonoid biosynthesis pathway.^35^ Overexpression of *OsMYBR22\OsRVE1* in rice (*Oryza sativa* L.) inhibited the expression of all examined chlorophyll biosynthesis-related genes including *protochlorophyllide oxidoreductase* A(OsPORA) *RA*).^36^ In contrast, *SlMYB72*, an activator transcription factor in tomato (*Solanum lycopersicum*), regulates chlorophyll accumulation by directly acting on protochlorophyllide oxidoreductase (POR) and Mg-chelatase H subunit genes (CHLH).^37^ In terms of growth and development, Huy et al. found that *MYB80* in *A. thaliana* can directly target glyoxal oxidase (GLOX1), pectin methylesterase (VANGUARD1), and A1 aspartic protease (UNDEAD) to further regulate pollen development and tapetum programmed cell death.^38^ In addition, in recent years, there have been endless reports on the response of MYB transcription factors to stress resistance. Yang et al. proved that DgMYB2 can directly target Glutathione peroxidase gene (*DgGPX1*) and increase the activity of GPX to reduce the accumulation of reactive oxygen species (ROS), thereby improving the cold resistance of *Chrysanthemum morifolium* Ramat.^39^ The relative electrolyte leakage (REL) and MDA in leaves of *OsMYB6* overexpressing rice were significantly reduced, indicating that *OsMYB6* gene may protect the integrity of plant cell membrane to cope with drought and salt stress.^40^ Transcriptome analysis showed that the *Medicago sativa.L* MYB transcription factor MsMYB4 significantly improved the salt tolerance of transgenic *A. thaliana* and *Alfalfa* in an ABA-dependent manner.^41^ MdMYB33 can bind directly to the MBS cis element on the *MdSOS1* promoter and regulate its activity, thereby promoting Na^+^ efflux and alleviating salt stress.^42^ Ectopic expression of *GaMYB85* can promote the accumulation of free Pro and chlorophyll in transgenic *A. thaliana*, as well as upregulation of stress-related genes such as Delta (1) – Pyroline-5 carboxylate synthase gene (*P5CS*), abscisic acid insensitive 5 gene (*ABI5*), and Class I alcohol dehydrogenase gene (*ADH1*), resulting in improved plant resistance to drought and salt stress.^43^ Zhang et al. also found that overexpression of *AtMYB49* in *A. thaliana* can increase the accumulation of Ca^2+^in leaves, reducing oxidative damage by upregulating the expression of antioxidant enzyme genes such as peroxidase gene (*POD*) and superoxide dismutase gene (*SOD*).^44^ In addition, MYB exerts its salt tolerance in a way that can act as an activator or inhibitor. Allogeneic expression of *Fagopyrum tataricum FtMYB9*^45^ and *FtMYB13*^46^ genes in *A. thaliana* enhanced drought tolerance and salt tolerance of transgenic plants. However, *FtMYB10*^47^ can also act as a suppressor of abiotic stress responses.

In summary, MYB protein plays a crucial role in plant response to salt stress. In this study, the effects of *MYB72* transcription factor on the antioxidant system, photosynthesis, and proline synthesis of *A. thaliana* under salt stress were studied through the joint analysis of physiology and transcriptomics, so as to further reveal the physiological and molecular mechanisms of *MYB72* response to salt stress.

## 2. Materials and methods

### 2.1. Plant materials and treatment

In this experiment, *A. thaliana* (‘ Columnbia-0 ’) was used as a control. *AtMYB72* overexpressing plants (OE-6, OE-10) were lines with high expression levels obtained by transgenic transformation in our laboratory. The mutants Salk108461 (*myb72*) and *myb72–1* were provided by Arabidopsis Biological Resource Center (ABRC). *A. thaliana* plants were cultured in a mixed medium of peat and vermiculite (1:1, V/V) for 3 weeks. The culture conditions were as follows: light intensity 150 μmol·m^−2^·s^−1^, humidity 50%, temperature 22°C, photoperiod 16 h/8 h (light/dark). Healthy seedlings with different genetic backgrounds of the same size were selected and irrigated with 100 mmol∙L^−1^NaCl, with the same amount of water as the control, 30 plants in each group. After 7 days of treatment, the phenotypic differences were determined for subsequent experiments.

### 2.2. Determination items and methods

#### 2.2.1. Chlorophyll content

The determination methods of Chla and Chlb refer to the experimental methods of Wellburn et al.^48^

#### 2.2.2. Determination of ROS content, antioxidant enzyme activity, and proline content

The kit produced by Suzhou Keming Biotechnology Co., Ltd. (Jiangsu, China) was used to determine the production rate of superoxide anion (O_2_^−•^), the content of proline (Pro) and hydrogen peroxide (H_2_O_2_), and the activity of superoxide dismutase (SOD), peroxidase (POD), catalase (CAT), ascorbate peroxidase (APX). Three biological replicates were set for each measurement.

#### 2.2.3. Determination of malondialdehyde, soluble sugar, and soluble protein content

The content of malondialdehyde (MDA) was determined by thiobarbituric acid method.^49^ The contents of soluble protein (SP) and soluble sugar (SS) were determined by Coomassie brilliant blue G-250 staining and acid ninhydrin colorimetric method of Bradford et al.^50^

#### 2.2.4. Determination of Fast chlorophyll fluorescence curve and fluorescence parameters

The leaves of *A. thaliana* under different treatments were treated in dark for 30 min. The Fast chlorophyll fluorescence (OJIP) curve and 820 nm light reflection curve (*MR*_820_) of the leaves after dark treatment were measured by Hansatech multifunctional plant efficiency meter (M-PEA, King ‘s Lynn, UK), and repeated for four times. The O, J, I, and P points on the OJIP curve corresponded to 0.01, 2, 30, and 1000 ms, respectively, and the corresponding time of K and L points was 0.3 and 0.15 ms, respectively. The maximum photochemical efficiency (*F*_v_/*F*_m_) and PSII performance index based on light absorption(*PI*_ABS_) were obtained by JIP-test analysis of OJIP curve with reference to the method of Strasserf et al.^51^ *F*_0_ is defined as 0, *F*_m_, *F*_J_ and *F*_K_ are defined as 1, respectively. According to the formula *V*_O-P_=(*F*_t_-*F*_0_)/(*F*_m_-*F*_0_), *V*_O-J_=(*F*_t_-*F*_0_)/(*F*_J_-*F*_0_) and *V*_O-K_=(*F*_t_-*F*_0_)/(*F*_K_-*F*_0_), the OJIP curves are standardized to obtain *V*_O-P_, *V*_O-J_ and *V*_O-K_ curves. The *V*_O-P_, *V*_O-J_ and *V*_O-K_ curves of OE-10 plants were compared with the standardized curves of Col-0 plants to obtain △*V*_O-P_, △*V*_O-J_ and △*V*_O-K_ curves. The relative variable fluorescence of J point on *V*_O-P_ curve, K point on *V*_O-J_ curve and L point on *V*_O-K_ curve are represented by *V*_J_，*V*_K_ and *V*_L_, respectively. The activity of the PSI reaction center is represented by the slope △*I*/*I*_o_ of the initial segment of the MR820 curve. *I*_o_ and △*I* represent the maximum value of the reflected signal in the MR820 curve and the difference between the maximum and minimum values.^52^

#### 2.2.5. RNA-seq

Arabidopsis leaves from different treatments were cut into strips, wrapped in tin foil, frozen in liquid nitrogen for 1 h, and then all samples were sent to Wuhan BGI Sequencing Co., Ltd. in China for transcriptome analysis. RNA-Seq (Quantification) based on next-generation high-throughput sequencing technology is used to study gene expression and provide accurate digital expression profiles by sequencing and comparing transcripts. The sequence data were filtered with SOAPnuke (v1.5.2) (https://github.com/BGI-flexlab/SOAPnuke) and the clean reads were mapped to the reference genome using HISAT2 (v2.0.4) (http://www.ccb.jhu.edu/software/hisat/index.shtml). The clean reads were aligned with the reference gene set by Bowtie2 (v2.2.5) (http://bowtiebio.sourceforge.net/%20Bowtie2%20/index.shtml), and the gene expression level was calculated by RSEM (v1.2.12) (https://github.com/deweylab/RSEM.) said 49-year-old Kyoko Encyclopedia of Genes and Genomes (KEGG) (https://www.kegg.jp/) enrichment analysis of expressed genes was conducted using Phyper. The significance levels of the KEGG terms and pathways were corrected using a Q-value with a strict threshold (Q-value ≤0.05).

### 2.3. Data processing

Excel (Office, 2019; Microsoft Corp., Redmond, WA, USA) and SPSS 24.0 (IBM Corp, Armonk, NY, USA) were used for the statistical analysis. Differences among treatment groups were compared using Single-factor analysis of variance and least significant difference (LSD) methods. Each parameter was determined in triplicate, and diagrams were generated using the Origin2019b software.

## 3. Results

### 3.1. Differentially expressed gene enrichment analysis

**Figure 1.**
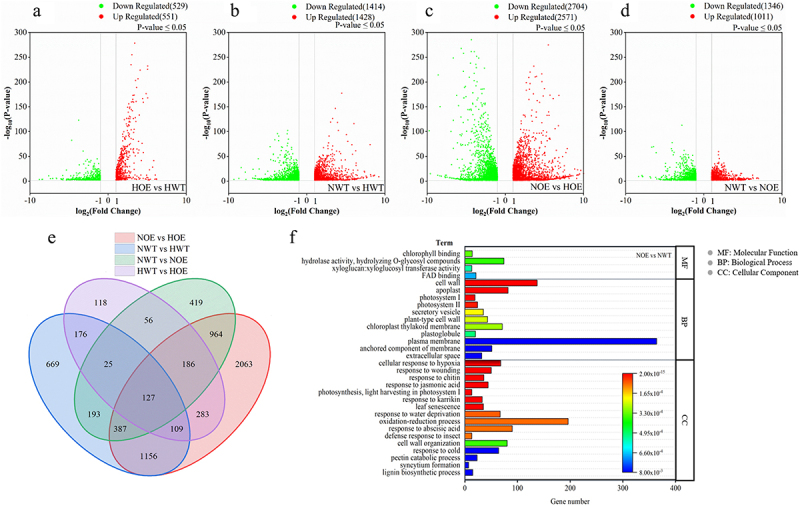
Volcano plot of DEGs (a，b，c and d), Venn diagram analysis results among different comparisons. (e), GO enrichment map (f). GO enrichment analysis selected GO terms with-logP adjust ranking in the top 32 for mapping.

Note: “HOEvsHWT”: overexpressed strain and wild-type strain under water treatment conditions; “NOEvsNWT”: overexpressed strain and wild-type strain under NaCl treatment; “HOEvsNOE”: overexpressed strain under water treatment and overexpressed strain under NaCl treatment; “HWTvsNWT”: wild-type strain under water treatment and wild-type strain under NaCl treatment.

As shown in Figure 1a, a total of 1080 differentially expressed genes (DEGs) were identified between OE-10 and Col-0 plants under non-stress conditions, of which 551 were up-regulated and 529 were down-regulated. Compared with CK, salt treatment increased the number of DEGs in Col-0 to 2842, including 1428 up-regulated genes and 1414 down-regulated genes (Figure 1b). The number of DEGs in OE-10 plants reached 5275, with 2571 up-regulated genes and 2704 down-regulated genes (Figure 1c). Through Figure 1a–c, the number of DEGs affected by salt stress in OE-10 plants was significantly higher than that in Col-0, especially the number of down-regulated DEGs. Two thousand three hundred and fifty-seven DEGs were identified between OE-10 plants and Col-0 plants under salt stress, of which 1011 were up-regulated and 1346 were down-regulated, which were significantly higher than those under non-stress conditions (Figure 1d). The results showed that there was a total of 6931 DEGs in the four comparison groups, of which 127 DEGs changed significantly in each group, accounting for only 1.83% of the total DEGs. There are 7305 unique DEGs in the 4 comparison groups, accounting for 47.16% of the total. In salt stress, the number of unique DEGs caused by *MYB72* overexpression was 2063, which was significantly higher than that in the CK group. Venn diagram analysis results showed that there were 1,779 common DEGs between NOE vs. HOE and NWT vs. HWT (Figure 1e).

Figure 1f shows that under salt stress, compared with Col-0, DEGs significantly affected by salt stress in OE-10 have 4 molecular functions, 11 biological processes, and 16 cellular components in GO term (*p <* .01). GO analysis showed that DEGs were mainly enriched in ‘cell wall’, ‘apoplast’, ‘photosystem I’, ‘photosystem II’ and other biological processes (*p <* .01). In terms of molecular function, ‘chlorophyll binding’, ‘FAD binding’, ‘hydrolase activity’ and ‘xyloglucosyl transferase activity’ were significantly enriched. GO terms were significantly enriched in the categories of ‘response to hypoxia stress’, ‘response to injury stress’, ‘response to chitin’, ‘response to jasmonic acid’, and ‘photosynthesis and PSI light-harvesting protein’ in the cell composition column (*p <* .01). These annotated results suggest that *MYB72* transcription factor may act as a key transcription factor in response to salt stress by influencing chlorophyll, photosynthesis, and oxidative damage.

### 3.2. Leaf morphology, chlorophyll content, and chlorophyll synthesis related gene expression

**Figure 2.**
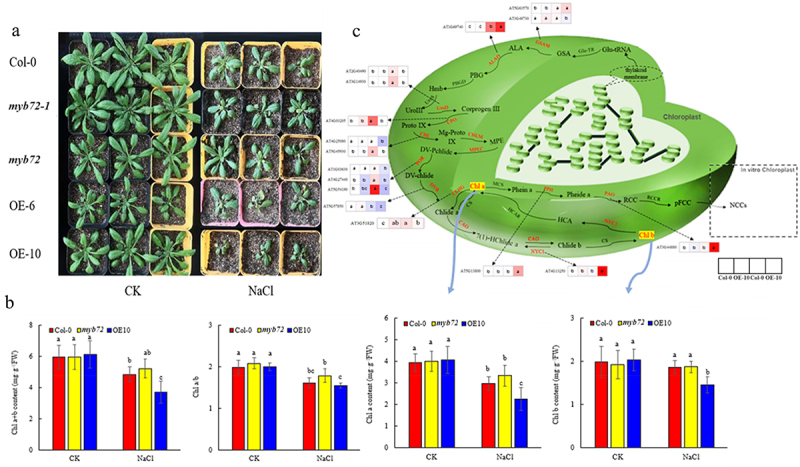
Effects of MYB72 overexpression on aboveground phenotype and chlorophyll metabolism under salt stress. (a) Phenotype of WT and transgenic lines under salt stress (b) chlorophyll a content, chlorophyll b content, chlorophyll a+b content, Chl a/b ratio (c) A. thaliana seedlings under NaCl stress and heatmaps of genes expression of chlorophyll synthesis and degradation. Note: The data in the figure is from three biological replicates (n = 3). Different lowercase letters indicate significant differences (*p* < .05). Abbreviations: Glu-tRNA: L-glutamyl-tRNA; Glu-TR: Glutamyl-tRNA reductase; GSA: L-glutamic acid 1- semialdehyde; GSAM: Glutamate-1-semi-aldehyde 2,1-aminomutase 1; ALA: δ-aminolevulinic acid; ALAD: Delta-aminolevulinic acid dehydratase; PBG: Porphobilinogen; PBGD: Porphobilinogen deaminase; Hmb: Hydroxymethylbilane; Uroporphyrinogen II; Uros: Uroporphyrinogen III synthase; UroIII: UroD: Uroporphyrinogen III decarboxylase; Coprogen III: Coproporphyrinogen III; CPO: Coproporphyrinogen-III oxidase; Proto IX: Protoporphyrinogen IX; CHL: Magnesium chelatase subunit H/D/I; Mg-proto IX: Mg-protoporphyrin IX; CHLM: Magnesium chelatase subunit M; MPE: Mg-protoporphyrin IX monomethyl ester; MPEC: Mg-protoporphyrin Ⅸ monomethylester cyclase; DV-Pchlide: Divinyl protochlorophyllide; DV-chlide: Divinyl chlorophyllide, POR: NADPH-protochlorophyllide oxidoreductase; Pchlide a: Protochlorophyllide a; DVR: Branched-chain-amino-acid aminotransferase; Chlide a: Chlophyllide a; CHLG: Chlorophyll synthase; Chl a: Chlorophyll a; CAO: Chlorophyllide a oxygenase; Chlide b: Chlorophyllide b; Chl b: Chlorophyll b; NYC1, chlorophyllide b reductase1; NOL, chlorophyllide b reductase; HCA: 7-hydroxymethyl chlorophyll a; HCAR1: 7-hydroxymethyl chlorophyll a reductase; PPH, pheophytinase; PAO, pheophorbide a oxygenase; RCC: Red chl catabolite; RCCR: red chlorophyll catabolite reductase.

As shown in Figure 2a, compared with normal growth conditions, the leaves of *A. thaliana* under salt stress showed different degrees of wilting, and the number and size of leaves decreased significantly. Among them, the salt damage degree of OE-10 was significantly higher than that of Col-0 and *myb72* mutant lines, accompanied by leaf yellowing. Under non-stress conditions, there was no significant difference in Chl a, Chl b, Chl a+b content, and Chl a/b ratio among Col-0, *myb72* mutant and OE-10 (*p <* .05). Salt stress led to a decrease in Chl a, Chl a+b content and Chl a/b (*p <* .05), while overexpression of *AtMYB72* significantly reduced Chl b content and Chl a/b ratio. In addition, under salt treatment, the content of Chl a, Chl b, Chl a+b, and Chl a/b ratio of OE-10 decreased by 24.5% (*p <* .05), 21.68% (*p <* .05), 23.41% (*p <* .05) and 3.6% (*p <* .05), respectively, compared with Col-0 (Figure 2b).

A total of 13 chlorophyll synthesis-related genes and 3 degradation-related genes were identified to be significantly changed (Figure 2c). Under CK conditions, except for *CHLG*(AT3G51820), the expression of other key enzyme genes in Col-0 and OE-10 was not significantly different. Compared to non-stress conditions, salt stress up-regulated most chlorophyll synthesis-related genes in Col-0 but had no significant effect on the expression of chlorophyll degradation-related genes. In addition, NaCl stress down-regulated the expression of *GSAM*(AT3G48730), *POR*(AT1G03630), *CHL*(AT4G25080) and *DVR*(AT5G57850) genes in OE-10 plants. At the same time, the expression levels of a few chlorophyll synthesis-related genes *GSAM*(AT5G63570), *ALAD*(AT1G69740), *CHLG*(AT3G51820) and chlorophyll degradation-related genes *PPH*(AT5G13800), *NYC1*(AT4G13250), *PAO* (AT3G44880) were significantly increased, and other genes did not change significantly. Compared with Col-0, except *ALAD*(AT1G69740), *CHLG*(AT3G51820) and chlorophyll degradation-related genes were significantly up-regulated, overexpression of *AtMYB72* gene significantly reduced the expression of chlorophyll synthesis genes under salt stress.

### 3.3. Chlorophyll fluorescence curve and parameters

**Figure 3.**
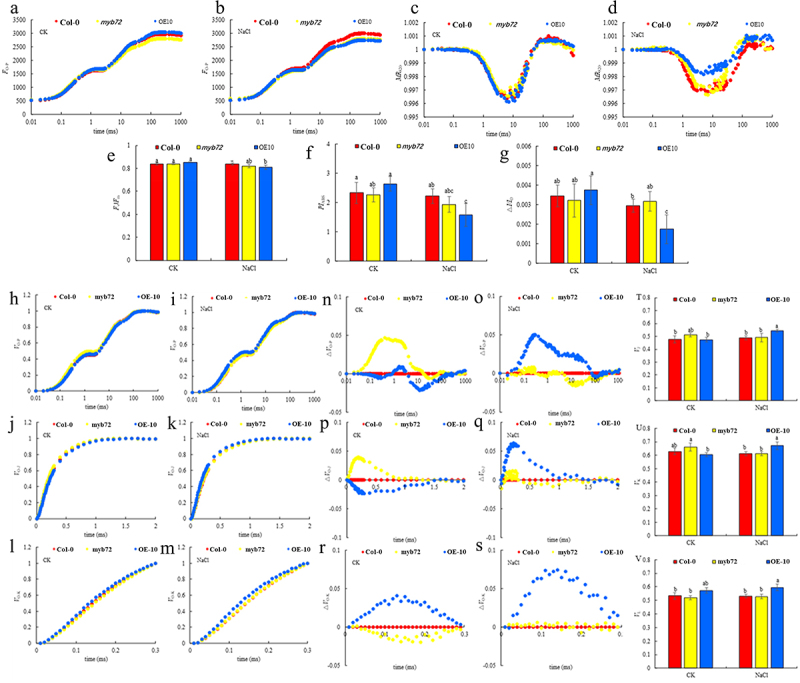
Effects of MYB72 overexpression on OJIP curves (a,b), MR820 curve (b), standardized O-P, O-J, and O-K curves (c, e and g), △V_O-P_，△V_O-J_ and△V_O-K_ curves (d, f and h), and Chlorophyll fluorescence parameters (i–s) of A. thaliana seedlings under NaCl stress.

Note: The data in the figure are from three biological replicates (*n* = 3). Different lowercase letters indicate significant differences (*p <* .05). (a) OJIP curves curve under CK and NaCl. (b) relative fluorescence curve under different treatments. (c) Δ*V*_O-P_=*V*_O-P_(treament)-*V*_O-P_(Col-0); (d) *V*_O-K_ is the fluorescence curve of points o-k, Δ*V*_O-K_(L-band) = (*V*_t_ − *V*_Col-0_)/(*V*_K_ −*V*_Col-0_), and so on; (h) o, j, i, p fluorescence intensity; (i) *F*v/*F*m, maximum fluorescence; (j) J point relative fluorescence intensity; (k) I point relative fluorescence intensity; (l) photosynthetic performance index; (m) total photosynthetic performance index; (n) The fluorescence intensity at 820 nm can indicate the activity of PSI; (o) quantify the difference between the lowest point and the highest point of MR820; (p) cyclic electron flow. Note: significant differences according to least significant difference (LSD) tests are indicated by different lowercase letters.

As shown in Figure 3a, under non-stress conditions, there was no significant difference in the OJIP curves of Col-0 and OE-10, and the relative fluorescence intensity 1(*F*_m_) of the P point on the *myb72* mutant plant curve was slightly reduced, but the *F*_v_/*F*_m_ and *PI*_ABS_ values of the three lines were not significantly different. Under stress conditions induced by NaCl treatment, there was no significant difference in the relative fluorescence value (*F*_O_) of Col-0, *myb72*, and OE-10 lines at O point. However, salt stress reduced the *F*_m_ of OE-10 more than Col-0. In addition, under salt stress, *F*_v_/*F*_m_ and *PI*_ABS_ of OE-10 were significantly lower than those of CK, while Col-0 and *myb72* mutants did not change significantly. Compared with Col-0, the relative fluorescence intensity (*F*_I_ and *F*_m_) of I and P points of OE-10 was significantly reduced after salt injury, and *F*_v_/*F*_m_ and *PI*_ABS_ also produced the same change, while *myb72* mutant had no significant difference (Figure 3a–f). Under non-stress conditions, there was no significant difference in the amplitude of *MR*_820_ curve and △*I*/*I*_o_ between OE-10 plants and Col-0 plants. Salt stress reduced the amplitude of the *MR*_820_ curve of Col-0, *myb72* mutant, and OE-10, especially in OE-10 plants, △*I*/*I*_o_ was significantly lower than Col-0 and *myb72* (Figure 3c,d,g).

The original OJIP curves were normalized to obtain *V*_O-P_, *V*_O-J_ and *V*_O-K_ curves (Figure 3h–m). Under non-stress conditions, the *V*_O-P_, *V*_O-J_ and *V*_O-K_ curves of Col-0, *myb72* mutants and OE-10 plants were less different, while NaCl treatment significantly increased the relative variable fluorescence(*V*_L_) of L points on the *V*_O-K_ curve of OE-10. The △*V*_O-P_, △*V*_O-J_ and △*V*_O-K_ curves were obtained by subtracting the *V*_O-P_, *V*_O-J_ and *V*_O-K_ curves of *myb72* and OE-10 from the standardized curves of Col-0, respectively (Figure 3n–s). In the △*V*_O-P_ curve, the relative fluorescence value of *myb72* at J point under CK condition was slightly higher than that of OE-10 and Col-0, while the △*V*_O-P_ curve of *myb72* and Col-0 under salt stress was similar, but the relative fluorescence value of OE-10 at J point increased and was significantly higher than that of *myb72* and Col-0, and the *V*_J_ quantitative results showed that it increased by 3.52% (*p <* .05). In the △*V*_O-J_ curve, it was found that the relative fluorescence values of *myb72* and OE-10 at 0.3 ms under CK conditions showed a decreasing and increasing trend compared with Col-0, respectively. Salt treatment caused OE-10 to have a positive peak at K point, namely K-band, while *myb72* and Col-0 had no significant difference. In addition, salt treatment resulted in a 10.4% increase in the *V*_K_ content of OE-10. On the contrary, under CK conditions, the relative fluorescence values of *myb72* and OE-10 at 0.15 ms of the *V*_O-K_ curve showed negative and positive peaks compared with Col-0, respectively, that is, L-band. After salt treatment, the relative fluorescence intensity of OE-10 at L point increased significantly, and the *V*_L_ quantitative results showed that it increased by 8.7%. In summary, overexpression of *MYB72* can significantly increase *V*_J_, *V*_K_, and *V*_L_ under salt stress.

### 3.4. Expression of light reaction related genes

**Figure 4.**
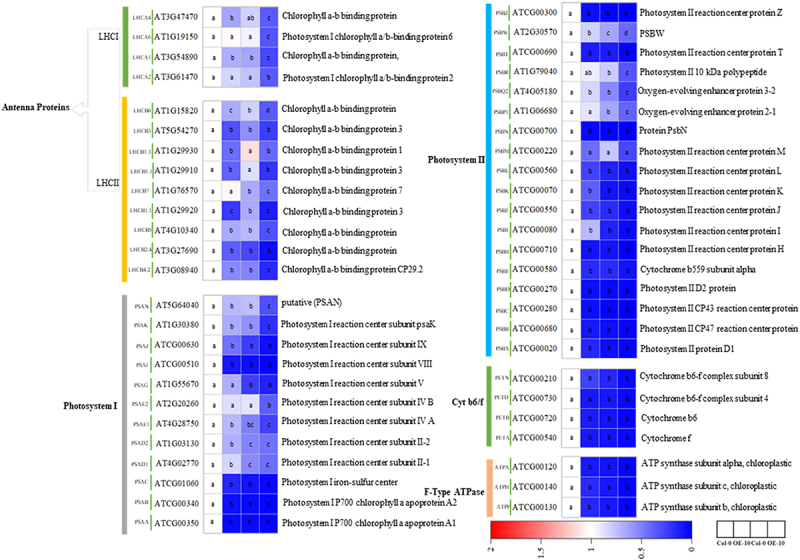
Heatmap of key genes expression of PSI, PSII, photosynthetic-antenna proteins, electron transfer, and ATP synthase in OE-10 and Col-0 A. thaliana seedlings under NaCl stress. Note: significant differences were expressed by different small letters (*p* < .05).

A total of 13 DEGs related to photosynthesis antenna proteins were identified. Under non-stress conditions, the expression of 10 related genes was significantly down-regulated in OE-10 plants compared with Col-0, including two PSI light-harvesting antenna proteins (LHCI) and eight PSII light-harvesting antenna proteins (LHCII) (Figure 4). Compared with the control, under NaCl stress, LHCI and LHCII genes of Col-0 and OE-10 plants were down-regulated except for LCHB3, LCHB1.1, LHCB1.3, and LCHB2.4, and gene expression of OE-10 plants was significantly lower than that of Col-0 plants. In addition, the two LHCI (LHCA2, LHCA6) genes were significantly decreased only in OE-10 plants, but had no significant changes in Col-0 plants under salt stress.

Under salt stress, 20 genes encoding PSI and PSII response centers in Col-0 and OE-10 plants (*psa*B, *psa*D, *psa*E, *psa*F, *psa*G, *psa*H, *psa*K, *psa*L, *psa*N and *psa*O, *psb*B, *psb*O, *psb*P, *psb*Q, *psb*R, *psb*S, *psb*W, *psb*Y, *psb*27, and *psb*28) have undergone significant changes. Under non-stress conditions, except *psa*E2, *psa*G in PSI and *psb*P1, *psb*W and *psb*M in PSII, the expression levels of the other 25 genes in OE-10 plants were significantly lower than those in Col-0 plants. After salt treatment, all genes except *psa*E2 and *psb*M were significantly down regulated by salt damage compared with control. Under non-coercive conditions, the expression levels of PSI-related genes *psa*N, *psa*K, *psa*E2, *psa*E1, *psa*D (*psa*D2, *psa*D1), PSII-related genes *psb*W, *psb*R, and oxygen evolution complex protein-related genes *psb*Q2 and *psb*P1 in OE-10 plants were significantly lower than those in Col-0 plants. In addition, four Cytb6/f complexes and three ATP synthase-related genes were identified, and their expression levels in OE-10 under non-stress conditions were lower than those of the wild type (*p* < 0.05).

### 3.5. ROS content, active oxygen scavenging enzyme activity and related gene expression

Under non-stress conditions, there was no significant difference in O_2_^−•^ production rate and H_2_O_2_ and MDA content among Col-0, *myb72* and OE plants. The increase of O_2_^−•^ production rate and MDA content under NaCl stress was significantly higher than that of CK group, but the content of H_2_O_2_ only increased significantly in OE-10. Under salt stress, the contents of O_2_^−•^, H_2_O_2_ and MDA in OE-10 were 32.08% (*p <* 0.05), 52.31% (*p <* .05) and 28.08% (*p <* 0.05) higher than those in Col-0, respectively, while the O_2_^−•^ in *myb72* mutant plants was significantly reduced by 21.09% (*p <* .05).

**Figure 5.**
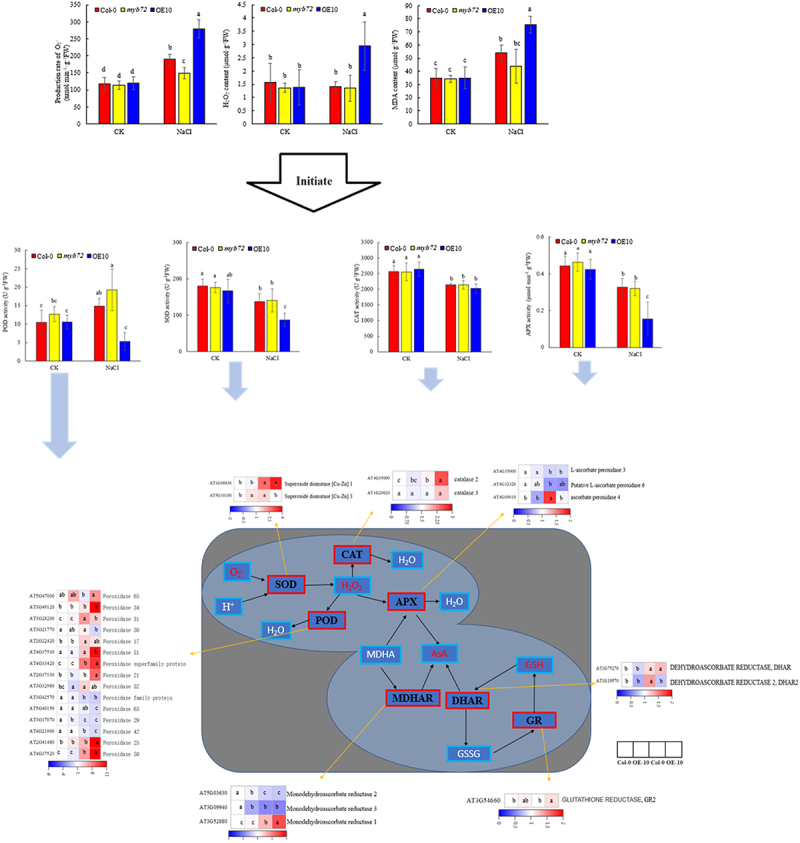
ROS content, ROS scavenging enzyme activity and gene expression map under salt stress. Note: significant differences were expressed by different small letters. O_2_^−•^: superoxide anion; H_2_O_2_: hydrogen peroxide. SOD: superoxide dismutase; POD: peroxidase; CAT: catalase; APX: ascorbate peroxidase; AsA: ascorbate; GSH: glutathione, GSSG: glutathiol; DHAR: dehydroascorbate reductase; DHA: dehydroascorbate; MDHA: monodehydroascorbate; MDHAR: monodehydroascorbate reductase; GR: glutathione reductase.

The increase of ROS content often causes the change of antioxidant enzyme activity. It can be seen from the figure that there was no significant difference in the activities of SOD, CAT, and POD between OE-10 plants and Col-0 plants under non-stress conditions (*p <* 0.05) (Figure 5). Compared with CK, salt treatment reduced the activities of SOD, APX, GPX, and CAT in *A. thaliana* leaves. It is worth noting that salt stress led to a significant increase in POD activity of Col-0 and *myb72*, which increased by 29.61% (*p <* .05) and 34.09% (*p <* .05), respectively, but decreased in OE-10 plants.

The expression analysis of key enzyme genes of AsA-GSH cycle showed that except *APX* (AT4G09010, AT4G32320), there were no significant differences in the expression levels of *APX*, *MDHAR*, *DHAR*, and *GR* genes between Col-0 and OE-10 plants under CK condition. Compared with the control, salt treatment significantly increased the expression levels of *APX* gene (AT4G09010) and *MDHAR* gene (AT3G52880) in Col-0 and OE-10 plants but significantly decreased the expression levels of two *APX* genes and *MDHAR* gene (AT3G09940, AT5G03630). The expression of Col-0 in OE-10 was more significant than that of Col-0. In addition, the expression of *DHAR*(AT1G75270) in OE-10 and wild type was significantly increased under salt treatment compared with control. The expression level of *DHAR*(AT1G19570) was significantly lower than that of wild type, while the expression level of *GR*(AT3G54660) was significantly higher than that of wild type.

### 3.6. Osmotic regulation substance content and gene expression of key enzymes in proline metabolism

**Figure 6.**
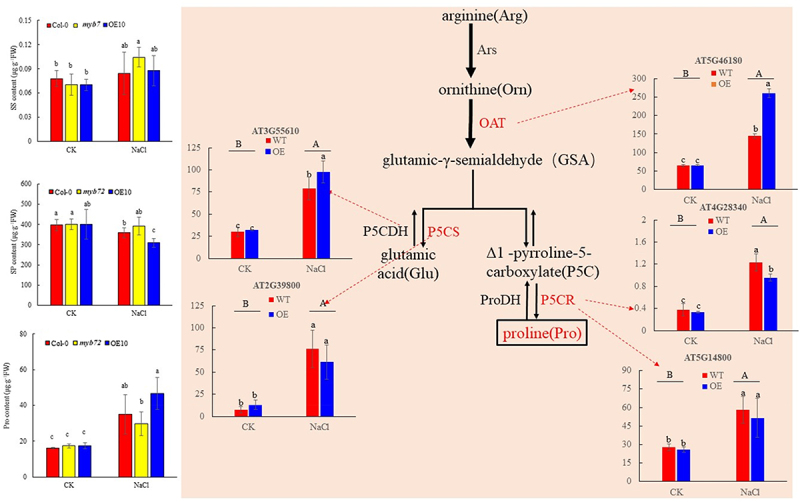
Effects of salt stress on soluble sugar, soluble protein and proline content, as well as changes in proline metabolism genes. Note: significant differences were expressed by different small letters. Glu :Glutamic acid; Arg: Arginine; Pro: Proline; GSA: Glutamic-γ-semialdehyde; GSADH: Glutamic-γ-semialdehyde dehydrogenase; OAT: ornithine aminotransferase; Orn: Ornithine; P5C: delta-1-pyrroline-5-carboxylate; P5CS: delta-1-pyrroline-5-carboxylate synthase; P5CDH: delta-1-pyrroline-5-carboxylate dehydrogenase; P5CR: delta-1-pyrroline-5-carboxylate reductase; ProDH: proline dehydrogenase.

Under normal conditions, there was no significant difference in SS, SP, and Pro contents among Col-0 plants, *myb72* mutant plants, and OE-10 plants. After salt treatment, SP content decreased significantly, while Pro content increased significantly, which was significantly increased by 53.96%, 41.85% and 62.58% compared with CK (*p <* .05). Compared with Col-0, SP content in OE-10 decreased more significantly. Transcriptome gene expression analysis showed that there was no significant difference in the expression level of Pro metabolism-related genes between overexpression and wild-type lines under CK conditions (*p <* .05). After NaCl treatment, the expressions of anabolic genes *OAT*(AT5G46180), *P5CR* (AT5G14800, AT4G28340), and *P5CS* (AT3G55610, AT2G39800) in Col-0 and OE-10 plants were significantly increased. Compared with Col-0, overexpression of *MYB72* gene significantly reduced the expression of *P5CR*(AT4G28340) in plants under salt stress, but *OAT*(AT5G46180) and *P5CS*(AT3G55610) were significantly increased (*p <* .05) (Figure 6).

## 4. Discussion

### 4.1. MYB affected plant phenotype and chlorophyll content under salt stress

The increase of salt concentration in soil will affect the growth and metabolism of plants. Excessive concentration may destroy the semi-permeability of the plasma membrane, leading to the breakdown of the interstitial ion homeostasis. It also reduces chlorophyll content and photosynthesis, eventually leading to reduced plant productivity or severe symptoms and death.^53,54^ As one of the most important photosynthetic pigments in all plants, chlorophyll can absorb light energy through the sun, thereby controlling the photosynthetic capacity of plants. Chlorophyll usually degrades under salt stress. Therefore, the pigment has been used as one of the key criteria for evaluating the salt tolerance of many plant species.^55^ In this experiment, salt stress made *A. thaliana* leaves smaller and yellower, significantly reduced Chl a, Chl a+b content and Chl a/b, but Chl b did not change significantly. Therefore, Chl a was more sensitive to salt stress than Chl b. Other studies have confirmed that exposure to 100 μmol·L^−1^ Cd or 500 μmol L^−1^ Zn stress significantly reduced the chlorophyll content of *A. thaliana* and *A. halleri*^56^; Total Chl, Chl a and Chl b concentrations in *Stevia rebaudiana* Bertoni decreased significantly after 16 days of 5 g·L^−1^ NaCl irrigation.^57^ Under neutral salt stress, the contents of total Chl and Chl a in *Robinia pseudoacacia* and *Fraxinus velutina* decreased with the increase of salt stress, and the change of Chl b was small.^58^ In general, more than 17 enzyme-coding genes are involved in the chlorophyll biosynthesis pathway in higher plants;^59,60^ Studies have shown that the expression of chlorophyll synthesis genes and the content of intermediate metabolites (protoporphyrin-IX, Mg-protoporphyrin-IX, and protochlorophyllide) were generally reduced in wild-type tobacco plants exposed to 150 mM NaCl;^61^ The expression of genes involved in chlorophyll biosynthesis (including *HEMA1*, *CHLH*, and *CAO*) in cucumber seedlings decreased due to salt stress, especially *CHLH*.^62^ In this study, compared with Col-0, the overexpression of *AtMYB72* gene significantly down-regulated chlorophyll synthesis genes such as *GSAM*(AT3G48730), *POR*(AT1G03630), *CHL*(AT4G25080), and *DVR*(AT5G18660) in *A. thaliana* leaves under salt stress, and the key enzyme genes of chlorophyll degradation *PPH* (AT5G13800), *NYC1*(AT4G13250) and *PAO*(AT3G44880) were significantly up-regulated, thus further promoting the degradation of chlorophyll. This indicates that the transcription factor *MYB72* plays a negative regulatory role in chlorophyll metabolism. The MYB superfamily is the largest member of all gene families in *A. thaliana*.^63^ The R2R3-MYB family transcription factors play an important role in regulating secondary biological metabolism and stress response.^64,65^ A lot of evidence has also shown the same results. Under cold stress, CaMYB306 up-regulated the chlorophyll catabolism genes *SlPAO* and *SlPPH* in pepper (*Capsicum annuum* L.),^66^ resulting in decreased chlorophyll content. Overexpression of *OsMYBR22/OsRVE1* inhibited the expression of all tested chlorophyll biosynthesis-related genes, including *OsPORA* in rice.^36^

### 4.2. Effects of MYB on photosynthetic system under salt stress

Photosynthesis is a fundamental process to maintain life on earth, which occurs in chloroplasts. The photoreaction of photosynthesis is driven by two major complexes of photosystem II (PSII) and photosystem I (PSI) in series, which use the captured light energy to stimulate the primary donor, resulting in linear electron transfer.^67^ The peripheral antenna proteins (LHCI, LHCII) of PSI and PSII are responsible for the capture of light energy and the driving force of photosynthesis. They are composed of chlorophyll a/b binding proteins and corresponding pigments.^68^ These chlorophyll a/b binding proteins in the photosystem are encoded by the LHCA (PSI) and LHCB (PSII) gene families, respectively. In this study, DEGs were significantly enriched in the description of molecular functions and biological processes such as chlorophyll binding, Photosystem I, and Photosystem II in GO analysis. Compared with Col-0, 11 of the 13 subunit genes that constitute the LHCA and LHCB complexes in OE-10 plants were down-regulated by salt stress. Among them, the expression of chlorophyll a/b binding protein CP29.2 in OE-10 was significantly reduced. To maintain the optimal photosynthetic activity of plants in a changing environment, the state transition mechanism plays an important role as a short-term light adaptation mechanism, mainly including two states, state I and state II. This process is through phosphorylation or dephosphorylation of the LHCII complex, making LHCII migrate between PSI and PSII, thereby regulating the ratio of light excitation energy absorbed between PSI and PSII. Among them, under state II conditions, phosphorylated LHCII, and additional unphosphorylated LHCII can bind to plant PSI at other sites, and all LHCII can transfer the excited energy to the PSI core.^69–71^ Combined with previous studies, a highly homologous phosphorylation site (Thr16) was identified in the CP29 protein of *A. thaliana* under state two conditions. This modification promoted the assembly of the LHCII-PSII super complex, and the lack of chlorophyll a/b binding protein CP29 prevented the formation of the complex.^72^ Kargul^73^ et al. suggested that a significant state transition occurred due to the instability of LHCII-PSII super complex dissociation following hyperphosphorylation of chlorophyll a/b binding protein CP29. They proposed that *MYB72* might regulate the state transition under stress conditions by reducing the expression of CP29, disrupting the excitation energy balance between the two photosystems.

Chlorophyll a molecules P700 and P680 become energized and negatively charged when exposed to light then then lose their energy when electrons return to the ground state, emitting some energy as fluorescence.^74^ As a nondestructive method, chlorophyll fluorescence is used to screen the direction of light energy absorbed in the photosynthetic membrane. Their changes during environmental stress can be used to judge the degree of damage to photosynthetic organs and visualize the disturbance in thylakoid membrane caused by stress.^75,76^ In this experiment, the decrease of *F*_v_/*F*_m_ and *PI*_ABS_ of OE-10 lines under salt stress was significantly greater than that of Col-0, indicating that *MYB72* transcription factor significantly reduced the PSII photochemical activity and NaCl tolerance of *A. thaliana*. To further analyze the effect of *MYB72* on PSII status under salt stress, we standardized the obtained original OJIP curve. The increase of *V*_J_ and *V*_K_ is a sign of the damage of electron transfer in PSII receptor side and the damage of OEC in PSII donor side, respectively.^77^ The results showed that most of the coding genes of PSII reaction center protein, PSI reaction center protein, Cytb6/f, and F-type ATPase were down-regulated by salt stress. The *V*_J_ on the normalized O-P curve in leaves overexpressing *MYB72* was significantly increased by salt stress, and the expression levels of psbW and *psbR* genes on the PSII receptor side were decreased in OE-10 plants. In *A. thaliana*, studies have shown that the amount of oxygen released in the psbR deletion mutant is significantly lower than that in the wild type. In the absence of DCMU, the deletion of psbR causes the electron transfer from Q(A) to Q(B) to be blocked.^78^ The above results indicated that overexpression of *MYB72* resulted in decreased expression of *psbW* and *psbR*, which made the electron transfer on the side of PSII receptor more seriously blocked after salt stress, and destroyed the structural stability and normal function of PSII. The *psbP* protein is a component of PSII, which binds to the lumen side of photosystem II together with *psbO* and *psbQ* to form an oxygen-evolving complex and mainly plays a role in supporting water oxidation. In this experiment, overexpression of *MYB72* increased the *V*_k_ value on the standardized O-P curve and decreased the expression levels of *psbP1* and *psbQ2*. These results showed that *MYB72* transcription factor can aggravate the damage to the PSII donor side oxygen-evolving complex (OEC) of *A. thaliana*, thereby increasing salt sensitivity. In addition, PSI is in the downstream of PSII in the photosynthetic electron transport chain. The decrease of its activity and stability not only affects the photosynthetic electron transport but also aggravates the damage of PSII.^79^ In this experiment, the amplitude of the MR820 curve (△*I*/*I*_o_) reflecting the number of photoactive PSI reaction centers in *A. thaliana* leaves were reduced by salt stress, which was most significant in OE-10 plants. The subunits of the PSI reaction center complex (*psaD-psaH*, *psaK*, *psaL*, *psaN*, *psaO*) play an important role in binding ferredoxin (Fd), interacting with plastocyanin and maintaining the structural stability of PSI (许大全编著 2013). Under salt stress, *psaN*, *psaE1*, *psaE2*, *psaD1*, *psaD2* and other genes were down-regulated, indicating that PSI was inhibited in structure and function, while overexpression of *MYB72* gene promoted the decrease of these genes. The above results indicate that *MYB72* inhibits plant photosynthesis by destroying light energy capture and electron transport of PSI and PSII.

### 4.3. The effects of MYB on ROS production, membrane lipid peroxidation, and antioxidant mechanism under salt stress

Salt stress can induce oxygen molecules to accept electrons from high energy levels to produce a variety of free radicals and non-free radical forms of reactive oxygen species, this process results in membrane peroxidation, causing severe damage to intracellular membrane structure, enzyme proteins, and significantly inhibiting plant growth.^80,81^ The results showed that when *T. aestivum* seedlings were exposed to 150 mM NaCl and 250 mM NaCl for 5 days, the contents of MDA and H_2_O_2_ increased by 63% and 116%, 78% and 108%, respectively.^82^ Siddiqui et al.^83^ found that compared with the control, the contents of MDA, H_2_O_2_ and the O_2_^−•^ production rate of tomato seedlings under salt stress(100 mM NaCl) increased by 116%, 198% and 263%, respectively. Recently, Ali et al. .^84^ observed an opposite phenomenon, MDA content decreased but H_2_O_2_ increased in Lycoris plants exposed to salt stress (150 mM NaCl). Compared with non-stress conditions, the levels of MDA, H_2_O_2_ and O_2_^−•^ did not differ significantly in the mutant after Nacl stress, but increased significantly in OE-10, and only O_2_^−•^ increased significantly in the wild type, indicating that OE-10 suffered greater oxidative damage than col-0 and *myb72*. Proline is a multifunctional molecule that accumulates at high concentrations under various abiotic stresses. It acts as both a penetrant and a free radical scavenger to protect cells from oxidative damage.^85^ The Orn pathway and the Glu pathway are two important pathways for the synthesis of Pro in plants.^86^ The former is converted into Pro by two consecutive reduction reactions catalyzed by P5CS^40^ and P5CR. The latter is converted into P5C by OAT, and finally synthesized by P5CR. MYB transcription factors play an important role in Pro accumulation. Studies have shown that overexpression of *GmMYB3a* in Soybean reduced physiological parameters, including soluble sugar, free Pro and chlorophyll content, and more Pro was accumulated in transgenic rice. Overexpression of *GaMYB85* can accumulate free Pro and chlorophyll in transgenic *A. thaliana* and increase the expression of stress-related genes such as *P5CS*.^87^ Our study showed that salt stress promoted the accumulation of Pro content. Compared with Col-0, although overexpression of *MYB72* down-regulated the Pro synthesis gene *P5CR* (AT4G28340) compared with wild type, the Pro content did not change significantly. In addition, the expression levels of the other two Pro synthesis genes *OAT* (AT5G46180) and *P5CS* (AT3G55610) were up regulated. Therefore, the mechanism of *MYB72* transcription factor regulating Pro metabolism is relatively complex and needs further study. In addition to the adaptation of plants to salt stress through osmotic adjustment substances, in order to alleviate the toxicity of ROS to plant cells, the antioxidant enzyme system will also be activated. SOD is the first line of defense of the plant antioxidant system, which can convert the accumulated superoxide molecules into oxygen and H_2_O_2_, and then reduce H_2_O_2_ to maintain redox homeostasis in plants through CAT, POD and AsA-GSH cycle or key enzymes in the Trx-Prx pathway.^88^ Ali et al.^89^ found that the use of 80 mM and 160 mM NaCl treatment reduced the enzyme activity of SOD, POD and CAT in maize, which was basically consistent with our experimental results. Our study found that the activity of antioxidant enzymes such as SOD and CAT was reduced by salt stress. In overexpression plants, the activity of SOD and POD decreased more significantly, and the content of soluble protein also decreased significantly. However, the expression levels of [*Cu-Zn*] *SOD3* and *CAT* genes showed an upward trend, which may be caused by protein inactivation caused by excessive salt ion concentration in plants. Salt stress resulted in a decrease in APX activity in the AsA-GSH cycle, and the expression levels of *APX* (AT4G32320) and *MDHAR* (AT3G09940, AT5G03630) genes were down-regulated. Overexpression of *AtMYB72* promoted this down-regulation, and the content of H_2_O_2_ was significantly increased in OE-10 lines. Therefore, *AtMYB72* may reduce the removal of H_2_O_2_ in *A. thaliana* under salt stress mainly by negatively regulating the expression of *APX* and *MDHAR*. Similarly, under salt stress conditions, POD activity is elevated, leading to the up regulation of several genes that are crucial for eliminating excess in plants. However, overexpression of *MYB72* significantly reduced POD activity and some POD-coding genes, especially AT4G21960 and AT5G40150, so *MYB72* can be used as an inhibitor of POD. In conclusion, overexpression of *MYB72* gene greatly reduced the ability of plants to scavenge reactive oxygen species under stress conditions by inhibiting antioxidant enzymes.

## 5. Conclusions

Salt stress led to the inhibition of photosynthetic electron transport chain in *A. thaliana*, and the activity of PSII and PSI decreased. The overexpression of *AtMYB72* aggravated the inhibition of photosynthetic capacity of *A. thaliana* under salt stress by down-regulating the synthesis genes in the process of chlorophyll metabolism, the related genes in the electron transport chain and up-regulating the chlorophyll degradation genes. *AtMYB72* also destroyed the ability of plant antioxidant system to scavenge reactive oxygen species under salt stress through the activity of various antioxidant enzymes and gene expression, resulting in oxidative damage. Compared with Col-0, *AtMYB72* reduced the expression of genes encoding enzymes associated with SOD, CAT and AsA-GSH cycles. This study provides ideas for the physiological and molecular mechanisms of *AtMYB72* as a negative regulator of salt resistance in *A. thaliana*, offering a new choice for cultivating salt-tolerant crops.
